# Relation between Burnout and Sleep Problems in Nurses: A Systematic Review with Meta-Analysis

**DOI:** 10.3390/healthcare10050954

**Published:** 2022-05-21

**Authors:** María José Membrive-Jiménez, José Luis Gómez-Urquiza, Nora Suleiman-Martos, Almudena Velando-Soriano, Tania Ariza, Emilia Inmaculada De la Fuente-Solana, Guillermo A. Cañadas-De la Fuente

**Affiliations:** 1 Red Cross Nursing Center, University of Sevilla, 41009 Sevilla, Spain; mariajose.membrive@gmail.com; 2 Faculty of Health Sciences, Campus Universitario de Ceuta, University of Granada, 51001 Ceuta, Spain; jlgurquiza@ugr.es; 3 Faculty of Health Sciences, University of Granada, 18071 Granada, Spain; norasm@ugr.es (N.S.-M.); gacf@ugr.es (G.A.C.-D.l.F.); 4 Andalusian Health Service, San Cecilio Clinical University Hospital, 18016 Granada, Spain; srtavelando@gmail.com; 5 Department of Educational Psychology and Psychobiology, Faculty of Education, Universidad Internacional de La Rioja (UNIR), 26006 Logroño, Spain; 6 Brain, Mind and Behaviour Research Center (CIMCYC), University of Granada, 18071 Granada, Spain; edfuente@ugr.es

**Keywords:** burnout syndrome, meta-analysis, nursing, prevalence, systematic review, sleep disorders

## Abstract

Burnout can affect nurses’ sleep quality. The aim of this study was to analyze the relationship between burnout syndrome and sleep problems in nurses. A systematic review with meta-analysis was performed. PubMed, CINAHL and Scopus databases were used. Some of the inclusion criteria were quantitative studies, in which the levels of burnout and sleep disorders were investigated in a sample of nurses using validated scales. A total of 12 studies were included. Sociodemographic variables did not influence the relation between burnout and sleep problems, except for being female. The environment and workplace violence, together with psychological traits and shifts, affect the probability of developing burnout and insomnia. The meta-analysis sample was *n* = 1127 nurses. The effect size of the correlation between burnout and sleep disorders was r = 0.39 (95% CI 0.29–0.48) with *p* < 0.001, indicating that the higher the level of burnout in nurses, the greater the presence of sleep disorders. The positive correlation between burnout and sleep disorders is a problem that must be addressed to improve the health of nurses. Developing turnicity strategies, using warmer lights in hospital units during night shifts and eliminating the fixed night shift could improve nurses’ working conditions.

## 1. Introduction

Burnout syndrome has been studied throughout history by many researchers. Authors have defined burnout as a psychological phenomenon [1] or as a clinical pathology [2]. However, the most used conceptualization describes burnout as an emotional response to chronic work stress with three dimensions [3]. Burnout is characterized by the appearance of emotional exhaustion (EE), understood as a progressive loss of energy; depersonalization (D), expressed as hostility towards the work environment; and feelings of low professional accomplishment (PA) or loss of self-confidence and demotivation [3,4,5].

This syndrome has a high prevalence among professionals who base their daily work on interpersonal relationships, either with coworkers or with clients [6,7]. Health sector employees are among the most affected by this problem, with nursing professionals being one of the most vulnerable populations to chronic work stress due to the high degree of relationship between nurses and patients and the strong emotional involvement that this requires [8]. Nurses also usually suffer from work overload [9] and difficulties reconciling work and family life [10].

These personal conflicts in the work environment lead to the appearance of occupational stress and burnout [11]. On the other hand, the consequences that arise from nurse burnout are numerous, such as absenteeism; lack of motivation; impaired work performance and communication between colleagues [12]; concentration difficulty; poor organization; increased errors; decreased patient safety; lack of energy; and feelings of frustration, anxiety, depression and insomnia [8]. These symptoms are often underestimated by professionals, who self-medicate instead of asking for help in medical or psychological consultation [13]. Furthermore, it has a strong impact on the quality of care [6].

Burnout can influence nurses’ sleep quality, and it has been identified as a cause of insomnia [8]. It is important to analyze the relation between burnout and sleep problems in nurses. Furthermore, the work of nurses has some characteristics that can increase sleep problems and generate an increased release of adrenaline [14]. Nurses work rotating shifts and extended hours, which can change the circadian rhythm, occasionally leading to abuse of caffeine and benzodiazepines, [13,15], resulting in nurses needing to use their days off and free time to recover lost sleep hours [16]. All these factors can lead to an increase in sleep onset and maintenance insomnia, along with alterations in sleep architecture and a decrease in the quality and quantity of sleep hours [17].

This scenario leads to insufficient sleep quality, which plays an essential role in emotional regulation and mental wellbeing [18], creating a cyclical problem underlying nursing work [19]. Burnout syndrome, among other problems, leads to the appearance of insomnia, which is caused by the presence of chronic stress [20].

Sleep plays a fundamental role in learning, memory consolidation and motor learning, as well as in the immune system and cardiovascular and liver metabolism [21]. Insufficient sleep or circadian alteration can trigger a decrease in cognitive function and mental performance [22], psychiatric morbidity [23], physical fatigue, decreased levels of attention and concentration, increased risk of accidents, slow complex coordination and a weakening of the cardiovascular autonomic response system [24]. Female nurses are also at higher risk of metabolic disorders and disrupted circadian rhythms, which can lead to diseases such as diabetes mellitus, obesity and important cardiovascular diseases, such as coronary artery disease or hypertension [25].

Due to the negative repercussions of burnout and sleep disorders in nurses (decreasing quality of care and patient satisfaction, as well as increasing mistakes in healthcare, among others) and in order to avoid such problems, it is important to analyze the relationship between these phenomena to establish possible interventions to improve both parameters and achieve better health for nurses. To the best of our knowledge, there is no similar study reviewing the literature about burnout and sleep problems in nurses or other healthcare professionals. Thus, the aim of the present study was to analyze the relationship between burnout syndrome and sleep problems in nurses. A meta-analysis was performed to clarify the direction and the real effect size of the correlation between these variables with a larger sample of nurses with a higher level of evidence.

## 2. Materials and Methods

A systematic review of the literature was performed following PRISMA recommendations (preferred reporting items for systematic reviews and meta-analyses) [26,27].

### 2.1. Search Strategy

CINAHL, PubMed and Scopus databases were consulted. The search equation, based on MeSH terms, was: “burnout AND nurs* AND sleep disorders”. The search was performed in September 2021. There were no restrictions on the date of publication.

### 2.2. Inclusion and Exclusion Criteria

The inclusion criteria were the following: quantitative studies investigating the levels of burnout and sleep disorders in a sample of nurses and studies using validated scales written in English or Spanish. The exclusion criteria were studies employing a mixed sample of professionals that did not provide independent data for nurses.

### 2.3. Study Selection Process

Two members of the team [details omitted for double-anonymized peer review] performed the search and study selection independently. In case of disagreement, a third researcher [details omitted for double-anonymized peer review] was consulted. For the selection, a reading of the title and abstract was executed first, followed by a full-text reading and, finally, a reverse search within the references of the included studies.

### 2.4. Data Extraction and Synthesis

A data collection table was prepared in Microsoft Word. The variables collected from the studies were: (a) authors; (b) year and country of publication; (c) characteristics of the sample, such as number of subjects included, age and sex; (d) study design; (e) aim of the study; (f) scores on the burnout and sleep scales; (g) relation between burnout and sleep disorders; and (h) level of evidence and degree of recommendation.

### 2.5. Critical Reading and Level of Evidence

A critical reading of the included studies was conducted using the STROBE checklist for observational studies [28] and the TREND checklist for quasi-experimental studies [29]. The levels of evidence and grade of recommendation proposed by the Levels of Evidence Working Group of the Oxford Centre for Evidence-Based Medicine (OCEBM) [30] were used.

### 2.6. Data Analysis

A descriptive analysis of the information from the included studies was performed for the systematic review. StatsDirect software was used for the meta-analysis. A random-effect meta-analysis based on the correlation (r) effect size between burnout and sleep disorders was performed. For calculation of the correlation effect size, the sample size and the correlation between burnout and sleep were used. The correlation meta-analysis package in StatsDirect was used. A sensitivity analysis was performed, and publication bias was assessed with Egger’s linear regression. The I2 index was used as a measure of heterogeneity. Information related to the research materials can be accessed through the meta-analysis research data file.

## 3. Results

### 3.1. Search Results

**Figure 1:**
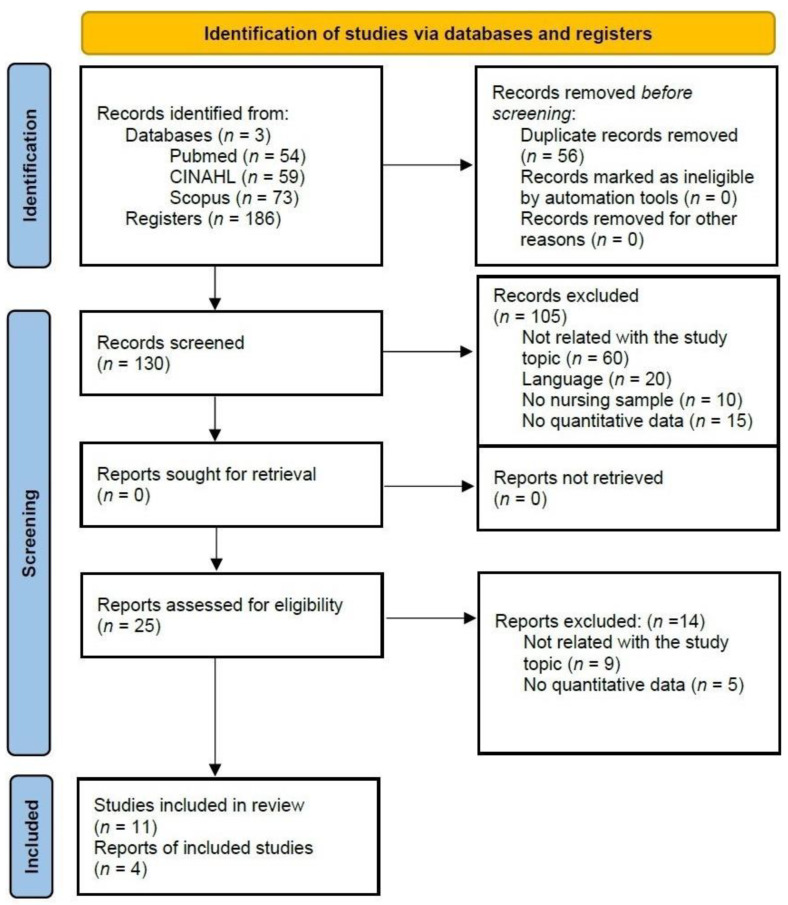
Flow diagram of search process.

A total of 186 articles were identified in the databases. After eliminating duplicates, 130 studies remained. Once the inclusion criteria were applied, *n* = 12 articles were selected for the systematic review, and 4 studies included the necessary information for meta-analysis. The search and selection process are shown in Figure 1.

### 3.2. Study and Participant Characteristics

The total combined sample of all included studies was 26,509 subjects. Of the 12 included studies, two were quasi-experimental [31,32], and the rest were descriptive and cross-sectional studies [33,34,35,36,37,38,39,40,41,42]. Of the included studies, 70% were published between 2015 and 2020 [32,33,34,35,37,39,40,41]. Regarding the geographic location of the included studies, four were conducted in Europe [31,34,36,39], four in Asia [32,37,38,41], two in North America [35,40] and two in Muslim countries [33,42]. The average age of the nurses who participated in the selected studies was between 29 and 55 years old, and all the samples had a higher percentage of women (more than 60% in most cases). The most used scale to measure burnout was the MBI (*n* = 8) [32,33,35,36,38,39,40,42], and the most used scale to measure sleep disorders was the Pittsburgh Sleep Quality Index (*n* = 6) [32,34,36,37,39,42]; two studies additionally used the Epworth Scale [32,39]. The characteristics of the studies are summarized in Table 1.

### 3.3. Burnout Syndrome and Sleep Problems in Nurses

A proportion of 15% of the nurses who participated in the analyzed studies had high levels of burnout [33], which is similar to data reported in other studies [39,41]. Some studies [34,42] found a positive and significant correlation between nurses who suffered from high levels of burnout and those who presented daytime sleepiness, together with low subjective quality of sleep [34]. Additionally, these sleep problems were predominantly correlated with the EE dimension [34,42]. It was found that 9.2% of the nurses were classified within the “withdrawing/burned out” profile [40]. This group, as well as the “frustrated/burning up” profile, presented significantly higher probabilities of suffering from sleep disorders.

### 3.4. Sociodemographic Variables and Probability of Suffering from Burnout and Sleep Disorders

Most of the sociodemographic variables studied did not affect sleep quality [42]. On the other hand, it was found in other studies that men presented lower levels than women, in terms of suffering from burnout and work problems [33]. Being female and suffering from burnout were significantly associated with deterioration of sleep quality [34].

### 3.5. Sleep Problems, Burnout Syndrome and the Organization of Nursing Work

Regarding the cycles of discontinuous work shifts or poorly organized overtime, an association was found with the risk of suffering from higher levels of burnout [33]. On the other hand, other types of nursing shift schedules were studied in [36]. The authors showed that when comparing 12-h and 8-h shift workers, those with the longer shift had worse health, wellbeing and sleep quality, as well as a higher level of burnout.

### 3.6. Other Risk Factors: Work Environment, Workplace Violence and Psychological Factors

Numerous studies found abundant predictive factors for suffering from burnout, which were positively correlated with the health environment [41] exposure to workplace violence, self-efficacy, sleep disorders and mild transient ailments, such as headaches, low back pain, odynophagia, etc. [38]. The risk factors found in nurses’ work environment were habitually witnessing the death of patients, especially in the emergency department [41], the multiple psychological demands of the patients and the lack of support from coworkers [33].

Nurses who had experienced an incident in their work environment in the last month (*p* < 0.001) or suffered a biological accident, such as a puncture (*p* = 0.010), presented worse sleep quality [42]. Likewise, other authors found that suffering from burnout was a strong mediator of the association between sleep disorders and violence in the workplace.

Therefore, nurses who worked in healthier work environments had higher levels of burnout and sleep disorders when they witnessed a violent incident at work [35]. On the other hand, psychological factors, such as self-criticism, personality traits with a strong temperament, fear of making mistakes [41] and compassion fatigue, were defined as mediators of the cyclical relationship between suffering from burnout and insomnia [37].

### 3.7. Psychological Interventions to Improve Sleep and Burnout

Regarding the experimental studies, a rehabilitation program based on psychoeducation for stress management strongly improved physiology, together with the quality of sleep, and allowed for a reduction in burnout, in addition to a consequent decrease in physical daytime fatigue [31]. On the other hand, a personal self-care program based on changing night-time habits and stress management improved the global score on the PSQI scale [32], in particular the subjective sleep quality item, as well as a significant reduction in latency and sleep disorders. However, this intervention only achieved significant results for sleep problems and not for symptoms of depression, burnout or the quality of life perceived by nurses [32].

### 3.8. Meta-Analysis of the Relationship between Burnout and Sleep Problems

**Figure 2:**
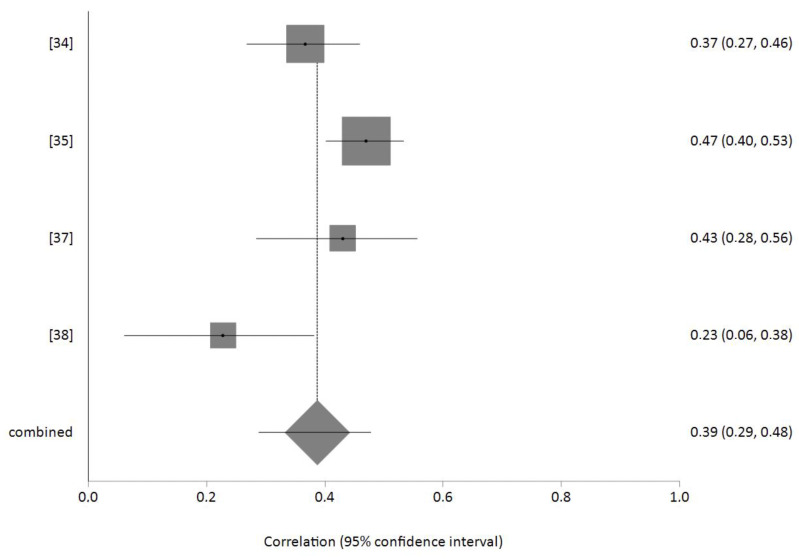
Forest plot of the relation between burnout and sleep problems.

Of the studies included in the systematic review, four included the necessary data for a meta-analysis of the effect size of the correlation between burnout and sleep problems. The total meta-analysis sample was *n* = 1127 nurses. The effect size was r = 0.39 (95% CI 0.29–0.48) with *p* < 0.001, indicating that the higher the level of burnout among nurses, the greater the presence of sleep problems. Egger’s test showed no publication bias, and the value of heterogeneity was I2 = 67.8%. A forest plot is shown in Figure 2.

## 4. Discussion

The aim of the study was to analyze the relation between burnout and sleep problems in nurses. With this study, we found a positive correlation between burnout and sleep problems, as well as some variables that may mediate this relation. Several studies included in the review established a positive correlation between nurses suffering from high levels of burnout, especially in terms of EE and low PA, with suffering from daytime sleepiness and low subjective sleep quality [34,39,40,42]. On the other hand, we identified an association between the different patterns of the circadian rhythm and the possibility of developing burnout based on stress tolerance and adaptation to the different shifts of each worker [33].

In the scientific literature, we find similar results, which explain the cause of this association. A chronic depletion of energy reserves, influenced by a constant activation of the hypothalamic–pituitary–adrenal axis and increasing levels of body stress, can generate burnout after a while, in addition to difficulty falling asleep and maintaining sleep [20]. Regarding the low personal accomplishment (PA) presented by nurses, there are multiple risk factors, both from the work environment and from the worker themself, with lack of training being one of the most influential factors [43], along with lack of support of the work team [44]. However, it has been shown that a good organization of resources in the work environment leads to greater job satisfaction, which acts as a protective factor against developing EE and increases workers’ PA [45,46].

Regarding the studies that analyzed the mediation of sociodemographic variables in the relationship between burnout and sleep disturbances [33,34,42], we found that the female sex is the main variable related to the probability of suffering from sleep disorders and burnout [34,42]. There are discrepancies in the scientific literature regarding this result. On the one hand, some authors have shown that women have greater difficulties in combining working hours in health centers, especially night shifts, with family life and motherhood, which contributes to greater stress in women, consequently affecting their quality of sleep, which was previously affected by raising children [47,48]. On the other hand, some authors state that male health professionals have a higher risk of developing burnout compared to women [49]. These data can be justified by the higher probability of men facing more responsibilities and conflicts on a daily basis at work [50,51].

Nursing professionals are subject to a specific work organization based on teamwork and distributed in shifts so that care for patients is covered 24 h a day, 7 days a week. This implies a cyclical, continuous or discontinuous rhythm of shifts with specific schedules [52]. In addition to the 8-, 10- and 12-h shifts, there is the possibility of performing extended shifts or additional physical hours due to service needs, increasing the daily work shift to a total of 24 h, with subsequent rest [53]. Different studies reflect the association between shifts of 12 h or more [36] and discontinuous or poorly organized work shifts with the presence of burnout [33], daytime sleepiness and sleep pattern disturbances [39]. The fixed night shift affects these variables the most, followed by the rotating shift [39]. Nurses who have rotating and night shifts sleep fewer hours per week than other fixed-shift workers who work during the day [19,54]. In addition, nurses who work night shifts and rotating shifts can suffer other health problems, such as varicose veins in the lower limbs, appetite disorder, alteration of leisure time [54] and stress, triggering greater professional burnout [55]. On the other hand, shifts longer than 8 h and with work overload strongly influence the probability of suffering high levels of EE, depersonalization and neuroticism [53].

In addition to developing sleep disorders [24,56] and daytime sleepiness, in many cases, days off could be considered insufficient to recover from such a high exposure to work stressors during extended shifts [36]. In many cases, it is impossible to change the work team’s organization; thus, it is necessary for these professionals to adopt measures to reduce variations in the sleep-watch rhythm [19], using a system of daily naps and adjusting their personal and family rhythms to their varying work schedules and days of payment [57]. Likewise, if the possibility exists, nurses can structure night shifts in a way that allows patients to be given quality care, including micro-naps lasting more than one hour, with the aim of presenting a greater recovery after the work shift [58].

The predictive factors of burnout and some sleep disturbances are closely related to the type of work performed by nurses in terms of the work environment, suffering workplace violence or the psychological factors of each worker. Thus, factors associated with the work environment, whether due to working with dying patients, psychological demands, lack of support from colleagues or having experienced a biological accident, are classified as potentially stressful events for workers and have somatic repercussions [33,39,41,42]; these data are corroborated in the scientific literature. The impact of the physical environment, workload and ambiguity of nursing care, were shown to be the main risk factors for severe stress, which, in turn, is associated with sleep disorders and digestive problems [59]. Other authors found caring for dying patients and seriously injured children to be the most stressful events, with somatic repercussions for nurses [60].

Regarding workplace violence, nurses who work in healthier work environments present higher levels of burnout and sleep disorders when they witness violent incidents at work compared to nurses who work in more hostile environments [35,38], which can be explained according to the theory that nurses who work in more violent environments are used to suffering stressful incidents throughout their working hours and face such incidents without suffering repercussions in sleep patterns or additional stress [61].

Regarding the psychological factors that enhance the cyclical relationship between suffering from burnout and insomnia [37,41], we found other studies with similar results. In other research, sleep quality and daytime sleepiness were identified as the variables most related to anxiety/insomnia and somatic symptoms [62]. On the other hand, some studies corroborate the association between burnout and personality traits. EE and D dimensions are positively correlated with neuroticism, anxiety and depression but negatively correlated with agreeableness, conscientiousness, extraversion and openness. The PA dimension has a negative correlation with neuroticism, kindness, conscience and extraversion [6,63].

Another finding is the inclusion of psychoeducational interventions for managing stress and daytime fatigue in nurses, thus improving physiology and quality of sleep [32], in addition to mediating recovery from chronic burnout [31]. Previously, cognitive behavioral therapy was used to treat problems of severe insomnia, sleep efficiency, the number of awakenings and waking time after the onset of sleep [64], as well as interventions with calming music before going to bed, combined with brisk walks [65]. At present, there is an increase in research that includes, in burnout prevention therapies, the management of somatic symptoms entailed by chronic stress through education in mindfulness and resilience, presenting a more protective effect in conditions of work stress [22,66].

Regarding the limitations of this study, there are numerous articles that reported on the relationship between anxiety and depression with sleep disorders but did not include burnout measurements. Almost all the studies included in the review were cross-sectional, and most employed convenience sampling, which makes it difficult to establish causal relationships and increases in the risk of bias, making it difficult to extrapolate the obtained data. Finally, it is important to indicate that some of the studies did not include information about the duration of the sleep problems, and the age of the participants varied (which can influence burnout and sleep quality), which could influence the results. This fact, coupled with the considerable heterogeneity in the measurement questionnaires used to study sleep problems and their subjective quality, means that the results should be interpreted with caution. Future research should analyze how some interventions that work for reducing burnout in nurses, such as mindfulness, may also improve sleep quality by reducing burnout.

### Implications for Practice

The relationship between sleep and burnout must be taken into account to improve nurses’ working conditions. Developing turnicity strategies that limit alterations in circadian rhythm and improve sleep quality could be positive. Using warmer lights in hospital units during night shifts could also be valued, as white light seems to be an important factor for sleep quality. In addition, where possible, elimination of the fixed night shift should be considered due to its impact on circadian rhythm.

## 5. Conclusions

The results of this study and meta-analysis provide information about the relationship between burnout and sleep problems. High burnout levels are correlated with higher levels of sleep problems in nurses. Some variables can influence these problems, such as gender, shift type, work environment and workplace violence. Improving burnout in nurses should be a priority for nurse managers because doing so may help to improve sleep quality and the quality of care. The importance of healthcare workers was made clear during the COVID−19 pandemic; therefore ensuring the health or nurses is important in the future.

## Figures and Tables

**Table 1:** Main results included in the review.

Author, Publication Year, Country	Study Type	Sample (*n*)	Instrument for Burnout Measurement and Sleep Disturbance	Aim	Mean ± SD	Main Results	LE/GR
Bagheri et al. [33] Iran	Cross-sectional	*n* = 684 Female 575 (84%) Age group 30–40: 285 (41%)	MBI CTI	To investigate the relation between the circadian rhythm amplitude and stability, and occupational stress with Burnout Syndrome and Job Dissatisfaction among shift working nurses.	Circadian rhythm Amplitude: Vigorous: 513 (75.2%) Languid: 169 (24.8%) Stability: Rigid 523 (76.7%) Flexible 159 (23.3%) MBI (Low/moderate/high) EE: 178 (26.1%)/240 (35.2%)/264 (38.7%) DE 308 (45.1%)/167 (24.5%)/207 (30.4%) PA 345 (50.6%)/169 (24.8%)/168 (24.6%) Global: 43 (6.3%)/542 (79.5%)/97 (14.2%)	About 15% of the nurses suffered from high levels of burnout syndrome. Psychological demand and workplace support were significant predictors of BS and JD. Male nurses reported lower BS and higher JD. Irregular shift working schedule was also related to significantly higher odds of BS and JD. Nurses classified as languid experienced significantly more BS and JD than vigorous nurses.	2 b/B
Ekstedt et al. [31] Norway	Quasi-experimental	*n* = 39 CG: *n* = 16 12 women, Mean age 43 IG: *n* = 23 16 women, Mean age 44	SMBQ SQI	To investigate the role of sleep physiology in recovery from burnout. Intervention: IG underwent a multi-modal rehabilitation program, based on accepted Cognitive Behavioral Therapeutic methods with the aim of reducing stress.	SMBQ (l–7 = high) (Mean ± SD) Baseline CG: 1.7 ± 0.2 IG: 5.7 ± 0.2 Follow up CG: 1.8 ± 0.3 IG: 3.5 ± 0.3 SQI (l–6 = good) (Mean ± SD) Baseline CG: 5.0 ± 0.1 IG: 2.8 ± 0.2 Follow-up CG: 4.9 ± 0.1 IG: 4.3 ± 0.1	The intervention resulted in a strong improvement of sleep physiology, with recovery from burnout and a relation between improved sleep and a reduction in fatigue. It is suggested that impaired sleep continuity may be part of the fatigue component of burnout.	2 a/B
Giorgi et al. [34] Italy	Cross-sectional	*n* = 315 Female 67% Age group 41–50: 48%	CBI PSQI	To investigate the relationship between sleep disorders, burnout and job performance in a shift-work population of nurses.	Impaired sleep quality 164 nurses (52.1%) Presence of burnout: 99 nurses (31.4%)	Female gender and personal burn- out were significantly associated with impaired sleep quality, and there was a significant linear association between the quality of sleep and burnout (q = 0.367; *p* < 0.001). Sleep disturbance, daytime dysfunction and subjective sleep quality showed a significant positive correlation with the mean values of the total burnout score and its relative dimensions and was predominantly correlated with personal burnout.	2 b/B
Havaei et al. [35] Canada	Cross-sectional	*n* = 537 Female *n* = 506 (95%) Mean age 39	MBI-HSS CCHS	To examine whether work environment conditions moderate the mediating effect that burnout has on the relationship between workplace violence and musculoskeletal injuries, sleep disturbances/insomnia and anxiety disorders.	Mean sleep disturbances/insomnia (Range 0–6) 3.3 ± 1.9 EE (range punctuation 0–54) 28.2 ± 13.3	Burnout mediated the relationship between workplace violence and sleep. In healthier work environments, workplace violence was more strongly related to increased reports of burnout and sleep disturbances compared to less healthy work environments.	2 b/B
Iskera-Golec et al. [36] Poland	Cross-sectional	*n* = 126 Group of nurses 12 h shift: *n* = 96 Mean age: 25 Group of nurses 8 h shift: *n* = 30 Mean age 26	MBI ESS PSQI	To compare measures of health, sleep, psychological and social wellbeing, job satisfaction and burnout of ICU nurses on 12-h and 8-h shifts.	MBI dimensions 8 h/12 h shifts EE (0–54 punctuation): 16.80/21.37 DE (0–30 punctuation): 9.27/7.43 PA (0–48 punctuation): 31.00/28.39 Sleep measures 8 h/12 h shifts General sleep disturbance (4–20 punctuation): 10.00/12.50 Tired after sleep (4–20 punctuation) 11.38/13.36 Premature awakening (4–20 punctuation) 10.33/9.28 Difficulties in falling asleep (4–20 punctuation): 9.35/8.72 Average length of sleep (h): 5.43/6.11 Ratio of average length of sleep to length of sleep declared as sufficient: 0.62/0.84	The 12 h shift nurses showed worse indices of health, well-being and burnout than the 8 h shift nurses. It is suggested that this may be associated with their longer daily exposure to the stress of work.	2 b/B
Kim & Na [37] Korea	Cross sectional	*n* = 140 Female 100% Mean age 30	ProQOL PSQI	To identify the relationships between various factors, including compassion, fatigue, satisfaction, depression, anxiety and sleep disorders among oncology nurses.	Burnout (mean ± SD) (range 10–50) 30.53 ± 4.69 High group (range 56 ≤ T) 35 (25.0%) Medium group (44 ≤ T < 56) 73 (52.1%) Low group (T < 44) 32 (22.9%) Sleep disorder (range 0–21) 9.66 ± 3.18	Compassion fatigue is composed of secondary traumatic stress and burnout. The levels of compassion fatigue showed significant positive correlations with depression, anxiety and sleep disorder.	2 b/B
Lu [38] Philippines	Cross-sectional	*n* = 135 Female 77% Mean Age 32	MBI Questionnaire Data Health and Illness	To explore the interaction between situational factors (the role stressors, hazard exposure and personal factors) and development of burnout.	Sleep disturbances: 57 subjects (42.2%) experienced once a day. Spearman correlations between sleep disorder and burnout was 0.228, *p* = 0.08	Regression showed factors associated with burnout were organizational role stress, hazard exposure, self-efficacy, age, number of working years, illness in the past 12 months, migraine, dizziness, sleep disorder, cough and colds, and diarrhea.	2 b/B
Moreno-Casbas et al. [39] Spain	A multicenter, observational, and descriptive study	*n* = 635 Female: 551 (87%) Mean age: 41	MBI MEQ ESS PSQI	To describe nurses’ perception in relation to the quality of care and their work environment. To explore nurses’ quality of sleep. To analyze the relationship between ward and work shift with nurses’ perception of their work environment, sleep quality and daytime drowsiness.	Burnout: High level, 86 nurses (15.4%) Low level 326 nurses 58.3% MBI scale high levels: EE 107 (17.8%) PA 148 (25%) DE 110 (18.4%) Epworth Scale: Excessive sleepiness 311(51.8%) Low sleepiness 166 (27.7%) Medium sleepiness 123(20.5%) PSQI (mean ± dt): Global score: 6.8 ± 3.387 Sleep quality: 1.35 ± 0.641 Sleep latency: 1.35 ± 0.957 Sleep duration: 0.76 ± 0.871 Sleep efficiency: 0.9 ± 1.026 Sleep disturbance 1.25 ± 0.481 Sleeping medication: 0.38 ± 0.827 Daytime disfunction: 0.72 ± 0.726 Sleepiness 0.72 ± 0.726	15.4% of the nurses had a high level of burnout, and 58.3% had low burnout. Sleep quality was 6.38 for nurses working day shifts, 6.78 for rotational shifts and 7.93 for night shifts. Significant differences were found between subjective sleep quality score, sleep duration, sleep disturbances and daytime dysfunction.	2 b/B
Morimoto et al. [32] Japan	Quasi-experimental	*n* = 25 IG: 10 CG: 15 Female 22 (95%), Mean age 39 *n* = 84 (withdrew)	MBI (Japanese version) ESS (Japanese version) PSQI (Japanese version)	To examine the effectiveness of SHT in hospital nurses in Japan. Intervention: 90 min workshop covering sleep hygiene education and brief stress management.	PSQI (*n* = 84) Global score: 6.2 + −2.9 Sleep quality: 1.5 + −0.7 Sleep latency: 1.1 + 1.0 Sleep duration: 1.6 + −0.7 Sleep efficiency: 0.2 + −0.4 Sleep disturbance 0.7 + −0.5 Sleeping medication: 0.3 + −0.8 Daytime disfunction: 0.8 + −0.8 Sleepiness 8.8 + −4.5 MBI EE: 16.4 + 4.7 DE: 12.3 + −4.4 PA: 11.2 + −2.8 BASELINE (IG/CG) PSQI Global score: 6.5 ± 2.9/5.8 ± 3.3 Sleep quality: 1.3 ± 2.9/1.6 ± 3.3 Sleep latency: 1.5 ± 1.9/1.4 ± 2.2 Sleep duration: 1.9 ± 2.8/2.1 ± 3.3 Sleep efficiency: 0.0 ± 0.9/0.0 ± 1.1 Sleep disturbance 1.1 ± 2.4/0.9 ± 2.8 Sleeping medication: 0.0 ± 2.5/0.5 ± 3.0 Daytime disfunction: 1.1 ± 4.4/0.4 ± 5.1 Sleepiness 8.2 ± 8.9/8.9 ± 10.3 MBI EE: 15.6 ± 27.2/16.1 ± 31.7 DE: 12.6 ± 23.2/11.8 ± 27.0 PA: 14.6 ± 6.7/15.2 ± 7.8 FOLLOW UP (IG/CG) PSQI Global score: 5.3 ± 3.0/6.1 ± 3.4 Sleep quality: 1.2 ± 2.9/1.7 ± 3.3 Sleep latency: 1.3 ± 1.9/1.3 ± 2.2 Sleep duration: 1.8 ± 2.8/2.1 ± 3.3 Sleep efficiency: 0.0 ± 0.9/0.0 ± 1.1 Sleep disturbance 0.8 ± 2.4/0.8 ± 2.8 Sleeping medication: 0.0 ± 2.5/0.5 ± 3.0 Daytime disfunction: 0.7 ± 4.4/0.6 ± 5.1 Sleepiness 7.9 ± 8.9/8.1 ± 10.3 MBI EE: 15.0 ± 27.2/6.7 ± 31.7 DE: 12.7 ± 23.2/12.3 ± 27.0 PA: 13.0 ± 6.8/14.3 ± 7.8	SHT improved subjective sleep quality (global PSQI scores). Participants with sleep problems at pre-test in the SHT group showed a significant reduction in sleep latency and sleep disturbance, which was not observed in the control group. No significant improvement was found in the SHT group for sleepiness. No significant improvement was observed in either group for depressive symptoms, burnout and quality of life.	2 a/B
Schult et al. [40] USA	Cross-sectional	*n* = 23,339 Female: 51,312 (61%) Age group: 50–59 y.o: 31%	MBI HSS Burnout profiles All employee survey: health promotion	To provide a population overview of burnout profiles by occupation in a healthcare sector employee population and to investigate how burnout profiles relate to self-reported health behaviors and chronic conditions.	Burnout profiles Engaged 43.8% Unfulfilled 21.3% Striving/overextended 11.0% Frustrated/burning up 14.7% Withdrawing/burned out 9.2% Sleep disorders OR = 1.98; 99% CI [1.85, 2.12]	Employees in the “frustrated/burning up” and “withdrawing/burned out” profiles had significantly increased odds of sleep disorders. 9.2% of the nurses had a withdrawing/burned out profile	2 b/B
Wilson et al. [41] India	Cross-sectional	*n* = 51 Female: 71% Mean age: 28	MBI Disturbed sleep	To measure the degree of burnout in doctors and nurses working in the emergency medicine department of four tertiary care teaching hospitals in South India.	Moderate–severe burnout: EE 64.8% DP 71.4% PA 73.3% Disturbed sleep 31.4%	Degree of burnout among nurses was moderately high in the three components, and some of the identified predictors were criticism, disturbed sleep, short-tempered nature, fear of committing errors and witnessing death.	2 b/B
Zencirci and Arslan [42] Turkey	Cross-sectional	*n* = 483 Female: 100% Mean age: 30	MBI PSQI MEQ	To assess the relationship between sleep quality and demographic variables, morning–evening type and burnout in nurses who work shifts.	Mean PSQI value of nurses was 7.32 ± 3.42 PSQI value of 79.1% (*n* = 382) of nurses was ≥5. High EE (*p* < 0.001) and DP (*p* < 0.001), as well as PA decreased sleep quality (*p* = 0.001).	Most sociodemographic variables did not affect sleep quality. Participants with poor sleep quality had high burnout levels. Most nurses who belonged to a type that is neither morning nor evening had poor sleep quality. Nurses who experienced an incident worsening their sleep patterns (*p* < 0.001) and needlestick or sharp object injuries (*p* = 0.010) in the last month had poor sleep quality. The subjective sleep quality and sleep latency points of evening types within created models for the effect of burnout dimensions were high.	2 b/B

Note. BS: burnout syndrome; CBI: Copenhagen Burnout Inventory; CCHS: Canadian Community Health Survey; CG: control group; CTI: circadian type inventory; DP: depersonalization; EE: emotional exhaustion; ESS: Epworth Sleepiness Scale; GR: grade of recommendation; HSS: Human Service Survey; IG: intervention group; JD: job dissatisfaction; LE: level of evidence; MEQ: Morningness-Eveningness Questionnaire; MBI: Maslach Burnout Inventory; OCEBM: levels of evidence of the Oxford Centre for Evidence-Based Medicine; PA: personal accomplishment; ProQOL: professional quality of life; PSQI: Pittsburgh Sleep Quality Index; SD: standard deviation; SHT: self-help therapy; SMBQ: Shirom–Melamed Burnout Questionnaire; SQI: Sleep Quality Index.

## Data Availability

The data presented in this study is available by contacting the corresponding author.
